# Management of severe haemolytic anaemia due to residual small mitral paravalvular leak post-percutaneous closure: a case report

**DOI:** 10.1093/ehjcr/ytaa101

**Published:** 2020-04-30

**Authors:** Reda Abuelatta, Lamiaa Khedr, Ibraheem AlHarbi, Hesham A Naeim

**Affiliations:** Madina Cardiac Center, Khaled Bin Waleed Street, 42311-6167 Madina, Saudi Arabia

**Keywords:** Haemolytic anaemia, Paravalvular leak, Mitral regurgitation, Percutaneous closure, Vascular occluder, Case report

## Abstract

**Background:**

Haemolytic anaemia is a complication of paravalvular leak (PVL). The correlation between the size of the leak and the severity of haemolysis is unclear. Small leaks can cause severe haemolysis, whereas significant leaks may cause no haemolysis.

**Case summary:**

We report the case of a 40-year-old male who underwent mechanical mitral and aortic valve replacement 20 years ago. In the last 3 years, the procedure was repeated three times due to infective endocarditis. He presented with severe shortness of breath. A transoesophageal echocardiogram with three-dimensional surgical view showed that both discs of the mechanical mitral valve opened sufficiently but a severe PVL had occurred at the 9–12 o’clock position. The location of the mitral valve was abnormal, the sewing ring was inserted high at the mid-interatrial septum. The mechanical aortic valve functioned well. Closure of the transcutaneous PVL was accomplished with two percutaneously implanted devices, leaving a small leak in between. After closure, he developed haemolytic anaemia (haemoglobin: 6 g/dL, lactate dehydrogenase: 1896 units/L, reticulocyte count: 4.6%). He then received 16 units of packed red blood cells. He developed acute kidney injury and was started on haemodialysis. We then installed two additional devices to completely close the mild residual leak and another device to resolve the bidirectional transseptal defect. After 2 days, his renal function returned to normal and anaemia improved (haemoglobin: 9.1 g/dL).

**Discussion:**

Mild residual paravalvular leak can cause severe haemolytic anaemia that is correctable via percutaneous closure of the leak.

**Learning points**

- Haemolysis can be diagnosed by a serum lactate dehydrogenase level >460 U/L and any two of the three following criteria: blood haemoglobin <13.8 g/dL for males or <12.4 g/dL for females, serum haptoglobin <50 mg/dL, and reticulocyte count >2%.
- Mild residual paravalvular leak (PVL) can cause severe haemolytic anaemia that maybe correctable by percutaneous closure of the leak.
- Development of dedicated devices for PVL closure is needed to improve outcomes for percutaneous PVL closure further.

## Introduction

Paravalvular leak (PVL) caused by a space between the patient’s natural heart tissue and the replaced valve. The incidence of PVL is 2–10% occur in the aortic position and 7–17% in the mitral position.^1^ In 1–5% of patients, PVL may present with severe clinical consequences, including heart failure, haemolysis, and endocarditis, which require immediate intervention.^2^ When haemolytic anaemia appears after a technically successful percutaneous device closure, another surgery or repeat percutaneous intervention may be required.^3^ Early PVLs usually result from surgical techniques, whereas late PVLs usually result from endocarditis or resorption of annular calcifications.^4^ Major adverse events associated with percutaneous PVL closure at 30 days are 8.7%.^4^

New-onset haemolysis post-PVL closure due to residual device leakage often resolves within 6 months after endothelialization. Haemolysis due to residual leakage around the device may be severe and require transfusion, necessitating closure of the residual leak, device exchange, or device removal. This case required redo percutaneous closure. Haemolysis is indicated when the serum lactate dehydrogenase level exceeds 460 U/L and any two of the three following criteria are present: blood haemoglobin <13.8 g/dL for males or <12.4 g/dL for females, serum haptoglobin <50 mg/dL, and reticulocyte count >2%.^5^

## Timeline

**Table:** 

At 1998	At the age of 20 years, the patient underwent mechanical mitral valve replacement (MVR) and aortic valve replacement due to severe rheumatic heart disease
At January 2015	The patient presented with prolonged fever, diagnosed as infective endocarditis with large vegetation on the mechanical MV. The first redo MVR done
At February 2017	Second redo MVR due to the recurrence of infective endocarditis
At March 2018	Third redo MVR due to severe paravalvular leak (PVL) and progressive heart failure
In the last 3 months	He presented by an shortness of breath (SOB), New York Heart Association (NYHA) Class IV. There were no clinical or laboratory findings suggestive of endocarditis. Transoesophageal echocardiogram showed both MV discs were seen opening well, severe PVL, its vena contracta measured 1.7 cm
Admitted and PVL closure done	PVL device closure with two devices (8 and 10 mm ventricular septal defect devices) performed. A small residual leak in between both devices accepted as a good result with the improved pulmonary venous flow
After 6 h	Post-closure he develops severe haemolytic anaemia requiring 16 units packed red blood cells
Third day post-closure	We decide to close the residual leak and the atrial septal defect. Another two devices completely closed the mild remaining hole with device closure of a bidirectional transseptal defect
Two days later	The renal function came back to normal with the improvement of anaemia to haemoglobin of 9.1 g/dL
After 1 week	The patient discharged home
After 6 months	SOB improved to NYHA Class II, her haemoglobin stable at 9 g/dL and transthoracic echocardiogram showed normally functioning both mechanical valves

## Case presentation

**Figure 1:**
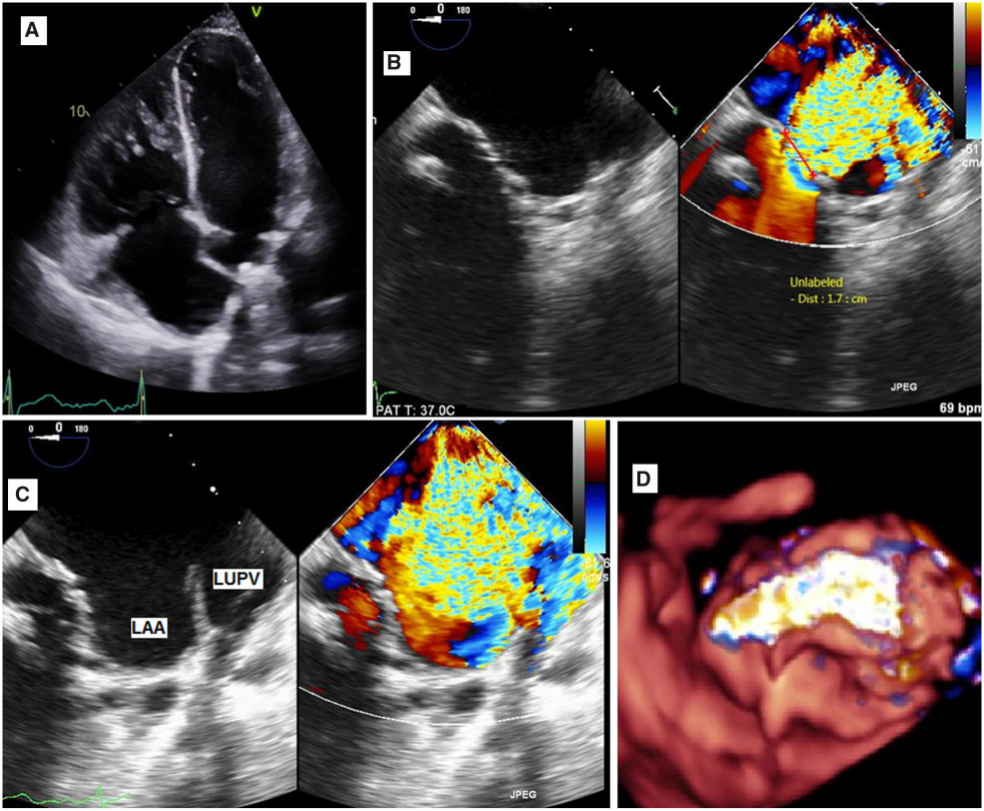
(*A*) Abnormally positioned mechanical mitral valve, its annulus at the mid-interatrial septum. (*B*) Severe paravalvular leak, vena contracta 1.7 cm. (C) Severe leak filled the left atrial appendage and left upper pulmonary vein. (*D*) Three-dimensional full volume with colour showed severe leak at 9 o’clock in surgical view. LAA, left atrial appendage; LUPV, left upper pulmonary vein.

A 40-year-old male presented with Class IV shortness of breath, according to the New York Heart Association (NYHA) functional classification, in the last 3 months. At presentation, blood pressure was 100/60 mmHg, heart rate was 105 b.p.m., respiratory rate was 24 cycle/min, and O_2_ saturation was 93%. There was a fine bilateral basal cripitations, elevated jugular venous pulse (JVP), and no lower limb oedema. In 1998, he underwent mechanical aortic valve replacement and mitral valve replacement (MVR) due to severe rheumatic heart disease. In 2015, he had a prolonged fever and was diagnosed with infective endocarditis, he underwent a mechanical MVR, which was repeated in 2017 due to recurrence of the endocarditis. In 2018, he had a fourth mechanical MVR due to severe PVL and progressive heart failure. He was on regular oral anticoagulation (Warfarin) with international normalized ratio around 3. The clinical and laboratory findings obtained upon admission did not suggest endocarditis. On examination, a pan-systolic murmur was heard over the left sternal border that radiated to the base of the heart and apex. A transoesophageal echocardiogram (TOE) indicated that the mechanical mitral valve was abnormally located, with its sewing ring inserted at the middle of the interatrial septum (*Figure 1A*). The distortion of the mechanical mitral valve anatomy was caused by the previous three mitral valve surgeries. Both mitral valve discs opened well; however, a severe PVL with vena contracta was measured at 1.7 cm (*Figure 1B*). The jet of the leak filled the left atrial appendage and the left upper pulmonary vein (*Figure 1C*). The leak was located laterally at the 9–12 o’clock position of the three-dimensional (3D) surgical view (*Figure 1D* and Supplementary material online*, Video S1*). The mechanical aortic valve was functioning well.

**Figure 2:**
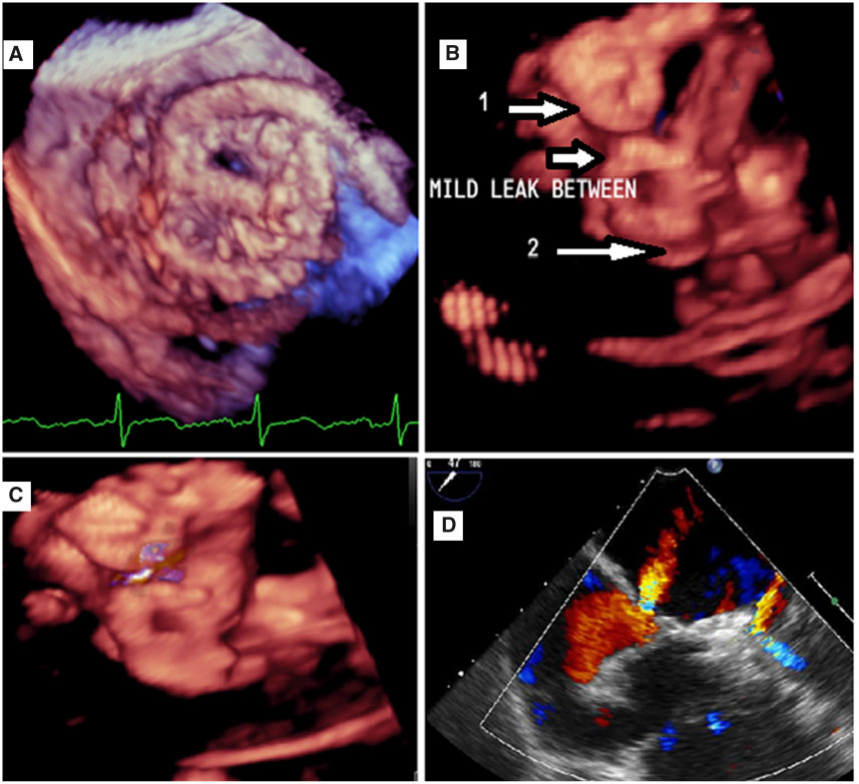
(*A*) Transseptal wire passing through the leak. (*B*) Two closure devices by three-dimensional zoom. (*C*) Full volume with colour showed mild leak between the two devices. (*D*) Mild residual leak, small bidirectional atrial septal defect at the site of the septal puncture.

We discussed the case in our heart team meeting and decided to perform a device closure of the leak. A Live 3D TOE was mandatory for guiding the procedure. The interventionist was dependent on the Live 3D while crossing the PVL, determining the type and size of the device, deploying of the device, and assessing the mechanical valve function after leak closure. The transseptal approach was selected because of the lateral position of the hole (*Figure 2A*). Transcutaneous PVL device closure was performed using 8 and 10 mm ventricular septal defect (VSD) closure devices. A small residual leak was left in between the two devices (*Figure 2B and C* and Supplementary material online*, Video S2*). The vena contracta of the residual leak was 2.5 mm, which was deemed acceptable due to the improved pulmonary venous flow.

At 6 h after closure, the patient became pale, jaundiced, and dyspnoeic with decreased urine output. The chest X-ray (CXR) showed mild congestion on small dose of diuresis. He developed severe haemolytic anaemia, diagnosed via laboratory data: (i) haemoglobin dropped from 10.0 to 5.9 g/dL, normal range is 12.4–14.9 g/dL. (ii) Serum lactate dehydrogenase increased to 1896 units/L, normal range is 122–222 units/L. (iii) Reticulocyte count increased to 4.6%, normal range is 0.5–1.5%.

**Figure 3:**
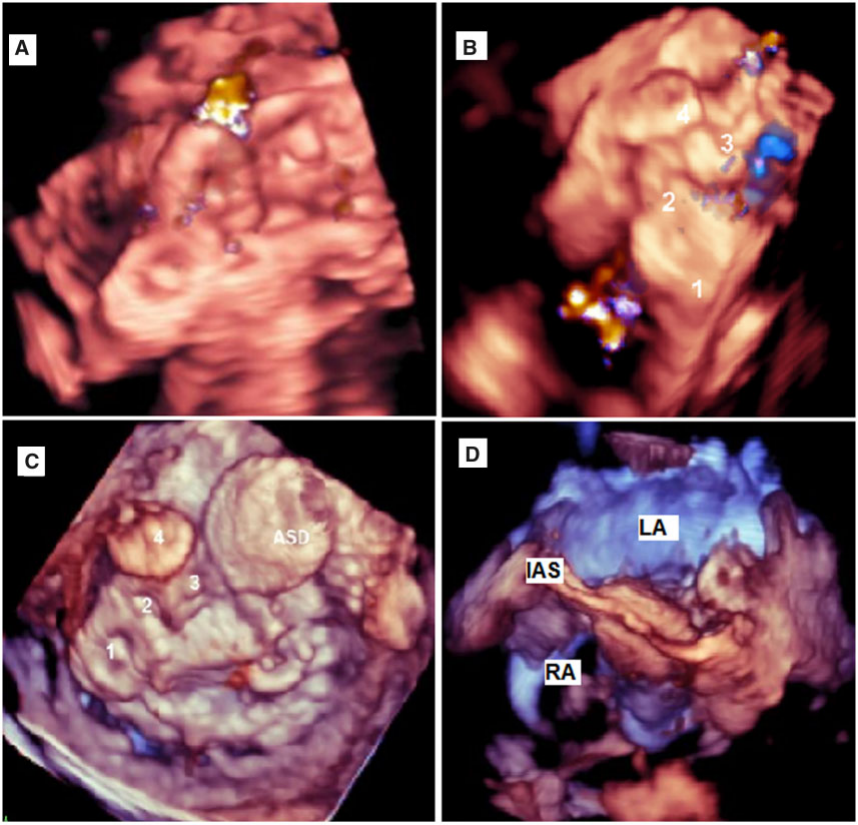
(*A*) Three closure devices, still mild leak. (*B*) Four closure devices, no leak. (*C*) Four closure devices plus 11 mm atrial septal defect closure device. (*D*) Both discs of atrial septal defect closure device showed. ASD, atrial septal defect; IAS, interatrial septum; LA, left atirum; RA, right atrium.

**Figure 4:**
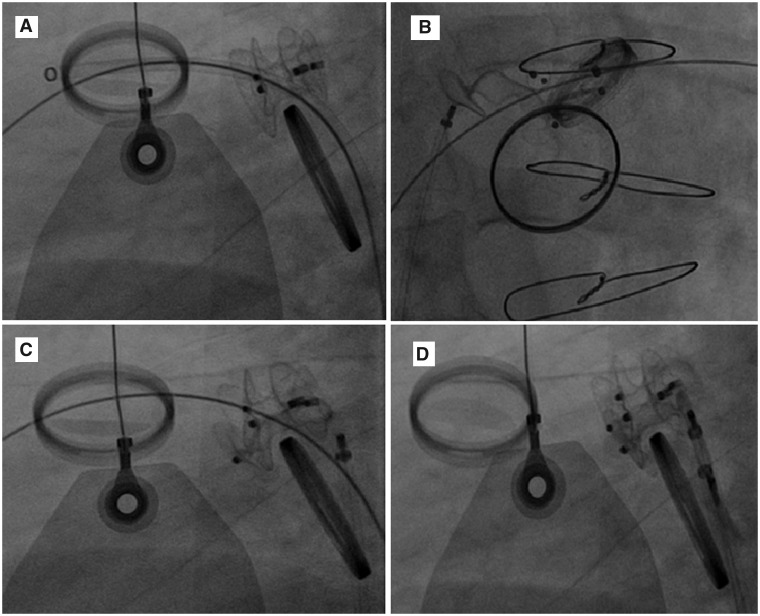
(*A*) Both mechanical mitral valve and aortic valve, two deployed closure devices. (*B*) Deployment of the third device. (*C*) Release of the third device. (*D*) Release of the fourth device. Note third and fourth devices deployed via apical approach.

He received 16 units of packed red blood cells after the leak closure. He developed acute kidney injury and was started on continuous renal replacement therapy. A small residual leak and a small atrial septal defect (ASD) with bidirectional shunt (*Figure 2D* and Supplementary material online*,Video S3*) were left during the PVL closure and we thought they are the cause of haemolysis. On the third day after the device closure procedure, we closed the residual leak and the ASD using the transapical approach to negotiate all the mitral valve aspects and to cross the small leak. An amplatzer vascular plug (AVP) II 8 mm device was deployed in between the two VSD closure devices. We detected a small leak beside the AVP II 8 mm device, we deployed another AVP II 8 mm device beside the first one, and the leak was no longer observed (*Figures 3A and B* and Supplementary material online*,Video S4*). Closure of a bidirectional interatrial septal defect with an ASD 8 mm device was performed (*Figure 3C and D* and Supplementary material online, *Video S5*). Although unlikely the ASD was contributing to haemolysis, we prefer to close it to avoid any flow turbulence beside the MV. The four closure devices were observed during fluoroscopy (*Figure 4* and Supplementary material online, *Video S6*). Two days after this procedure the patient’s renal function returned to normal, and his anaemia improved (haemoglobin: 9.1 g/dL). The patient was discharged after 1 week. At his 6-month follow-up appointment, his shortness of breath had improved to Class II (NYHA functional classification), his haemoglobin stabilized at 9 g/dL, and a transthoracic echocardiogram (TTE) showed normal function of both mechanical valves. The patient planned for regular follow-up every 6 months with TTE as needed.

## Discussion

One complication of PVL is haemolytic anaemia. The correlation between the size of the leak and the severity of haemolysis is unclear. Small holes can cause severe haemolysis, whereas significant leaks may cause no haemolysis. Smolka *et al*.^6^ analysed 116 patients with transcatheter PVL closure, he concluded that the PVL closure effectively reduced haemolysis if at least 90% reduction of PVL cross-sectional area was achieved. The effect was sustained in 6-month follow-up. Incomplete closure of PVL may increase the magnitude of haemolysis. In this case, the turbulent flow was due to a residual, small PVL. Closure of the leak effectively stopped haemolysis and led to clinical improvement. Almeida *et al*.^7^ reported a similar case using surgical replacement of the valve to cure the haemolysis. Joseph *et al*.^8^ reviewed techniques for percutaneous PVL closure and concluded that PVL closure is safe for most patients but associated with higher rates of residual leakage. Dedicated devices for PVL closure are needed to improve outcomes.

We recommend a trial of percutaneous reclosure of residual leaks to treat haemolysis, though more research is required to support this recommendation.

## Conclusion

Percutaneous closure of a small residual PVL may improve haemolytic anaemia. Development of dedicated devices for PVL closure is needed to improve outcomes for percutaneous PVL closure. For the high-risk symptomatic PVL patient, percutaneous closure is an alternative therapeutic strategy to surgical PVL repair.

## Lead author biography

Dr Hesham A. Naeim, MD, FASE, graduated from Faculty of Medicine, Al-Azhar University in December 1997. He was granted MSc degree in cardiovascular diseases in December 2002; granted MD degree in 2006. Diplomate—Adult Comprehensive Echocardiography from National Board of Echocardiography, USA in June 2014. He was a resident and assistant lecturer of cardiology, in Al-Azhar University hospitals from June 1997 to February 2006. He was a Cardiology consultant in Madina National Hospital Saudi Arabia from January 2007 till April 2013. He is an Adult cardiology consultant in Madinah Cardiac Center Saudi Arabia from June 2013 till now. He is expert in the field of echocardiography in structural heart disease.

## Supplementary material

Supplementary material is available at *European Heart Journal - Case Reports* online.

**Slide sets:** A fully edited slide set detailing this case and suitable for local presentation is available online as Supplementary data.

**Consent:** The author/s confirm that written consent for submission and publication of this case report including image(s) and associated text has been obtained from the patient in line with COPE guidance.

**Conflict of interest:** none declared.

## Supplementary Material

ytaa101_Supplementary_Data
