# Supplementary material for: Management of severe haemolytic anaemia due to residual small mitral paravalvular leak post-percutaneous closure: a case report
Source: Eur Heart J Case Rep. 2020 Apr 30;4(3):1–6. doi: 10.1093/ehjcr/ytaa101 (PMC7543880; doi:10.1093/ehjcr/ytaa101)
Supplement: ytaa101_Supplementary_Data [file ytaa101_supplementary_data.zip › ytaa101-suppl_data/EHJ-CR_Slide_Set.pptx]

## Slide 1
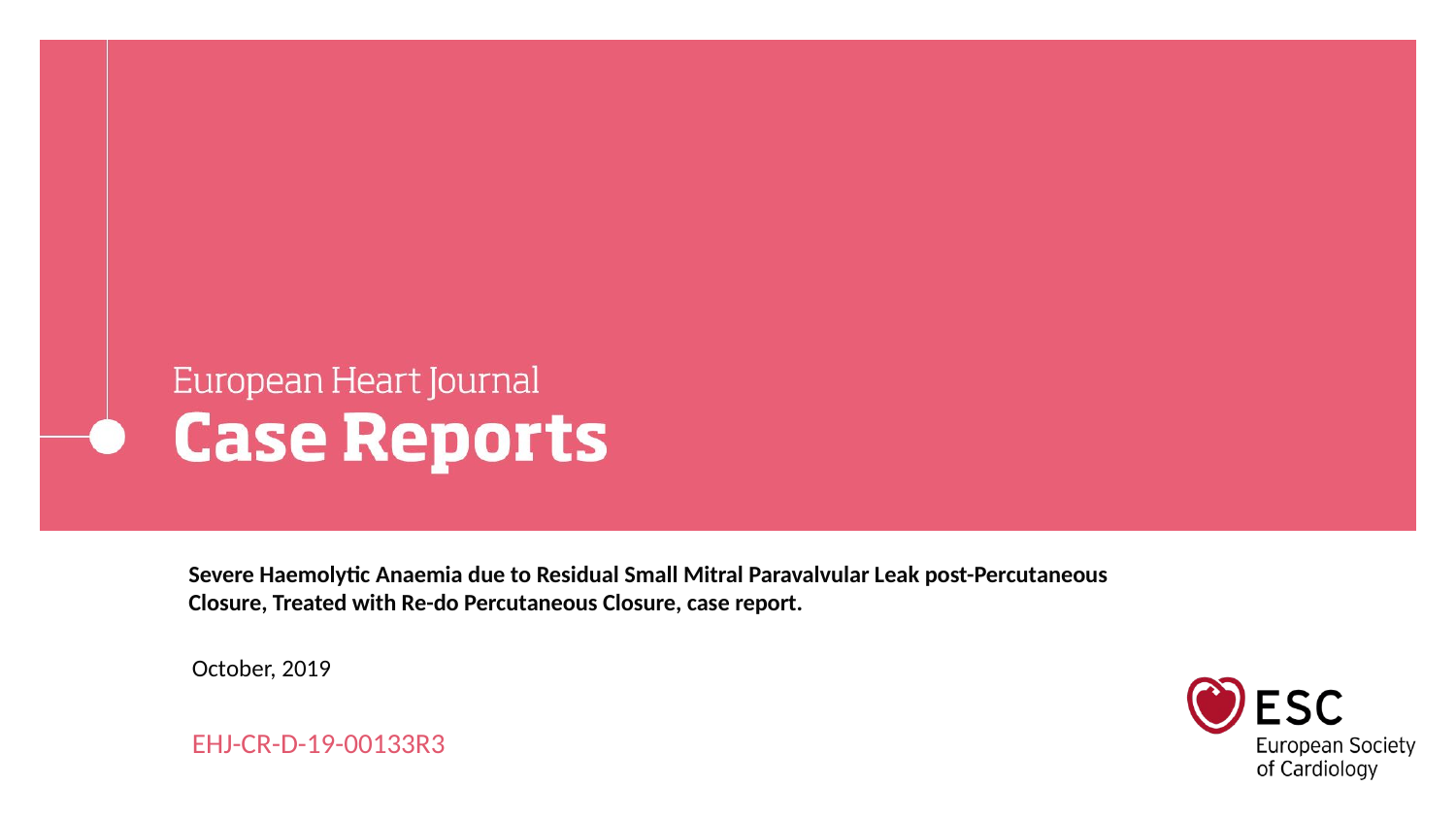

# Severe Haemolytic Anaemia due to Residual Small Mitral Paravalvular Leak post-Percutaneous Closure, Treated with Re-do Percutaneous Closure, case report.
October, 2019
EHJ-CR-D-19-00133R3

## Slide 2
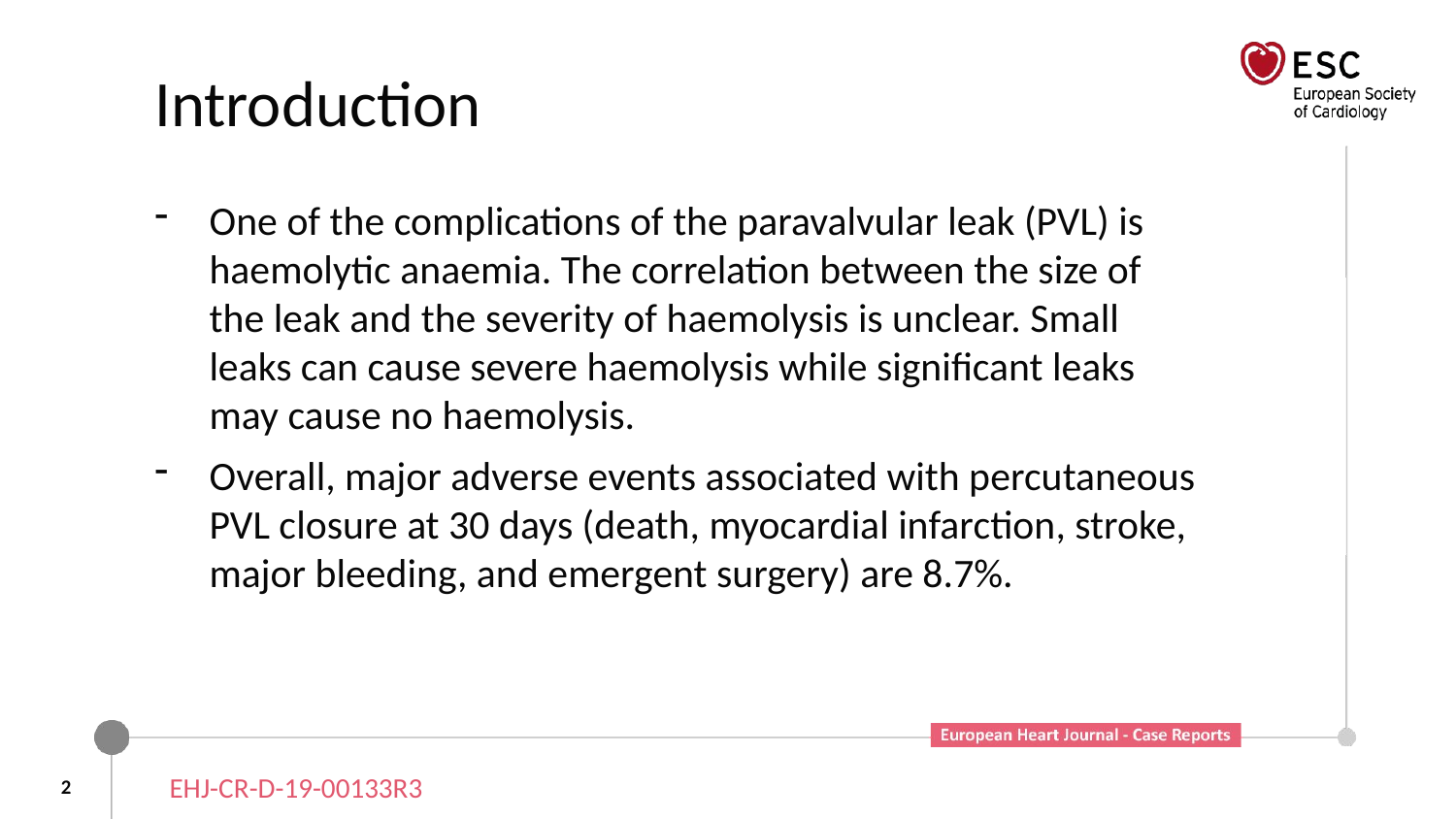

# Introduction
One of the complications of the paravalvular leak (PVL) is haemolytic anaemia. The correlation between the size of the leak and the severity of haemolysis is unclear. Small leaks can cause severe haemolysis while significant leaks may cause no haemolysis.
Overall, major adverse events associated with percutaneous PVL closure at 30 days (death, myocardial infarction, stroke, major bleeding, and emergent surgery) are 8.7%.
2
EHJ-CR-D-19-00133R3

## Slide 3
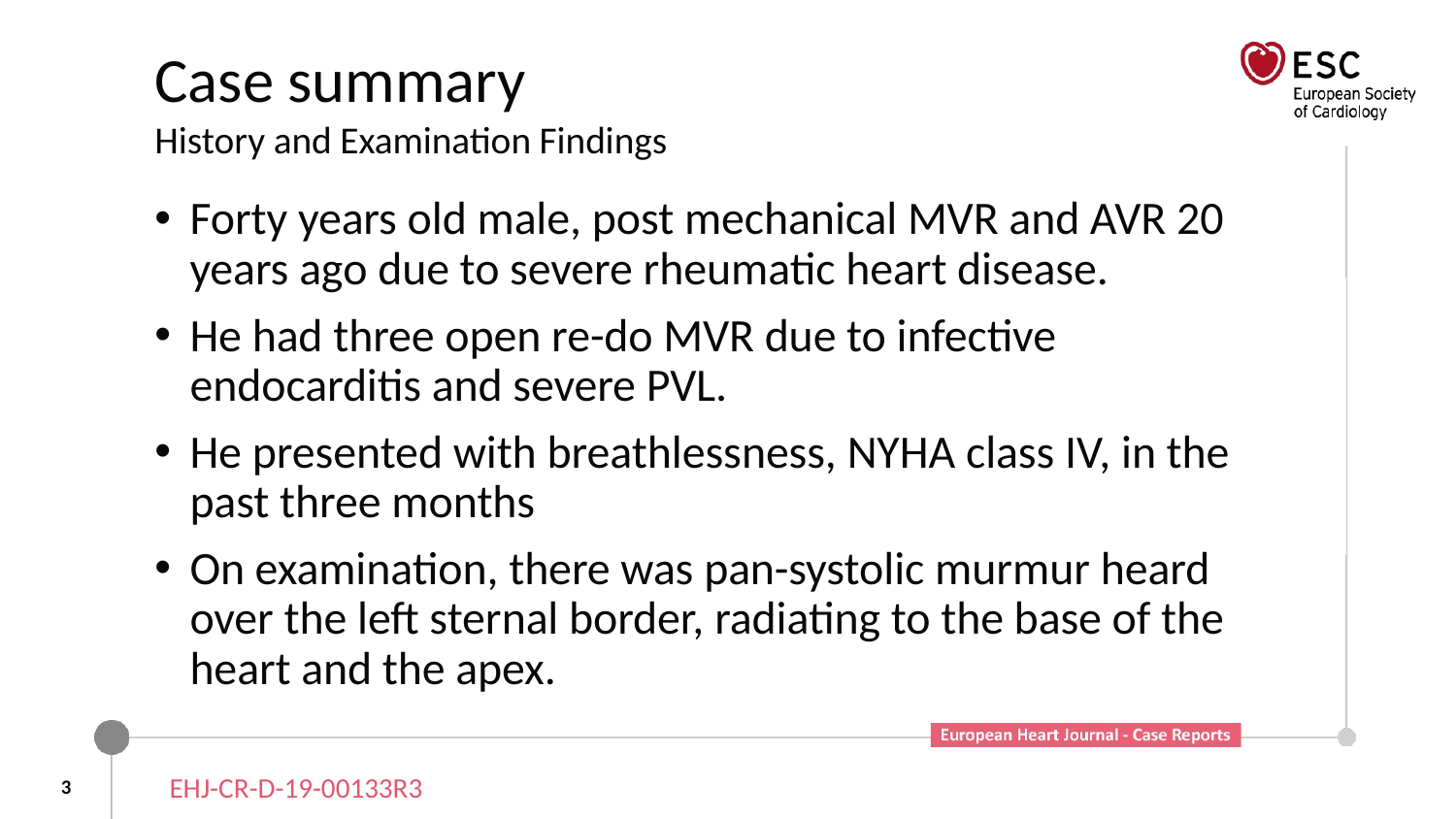

# Case summary History and Examination Findings
Forty years old male, post mechanical MVR and AVR 20 years ago due to severe rheumatic heart disease.
He had three open re-do MVR due to infective endocarditis and severe PVL.
He presented with breathlessness, NYHA class IV, in the past three months
On examination, there was pan-systolic murmur heard over the left sternal border, radiating to the base of the heart and the apex.
3
EHJ-CR-D-19-00133R3

## Slide 4
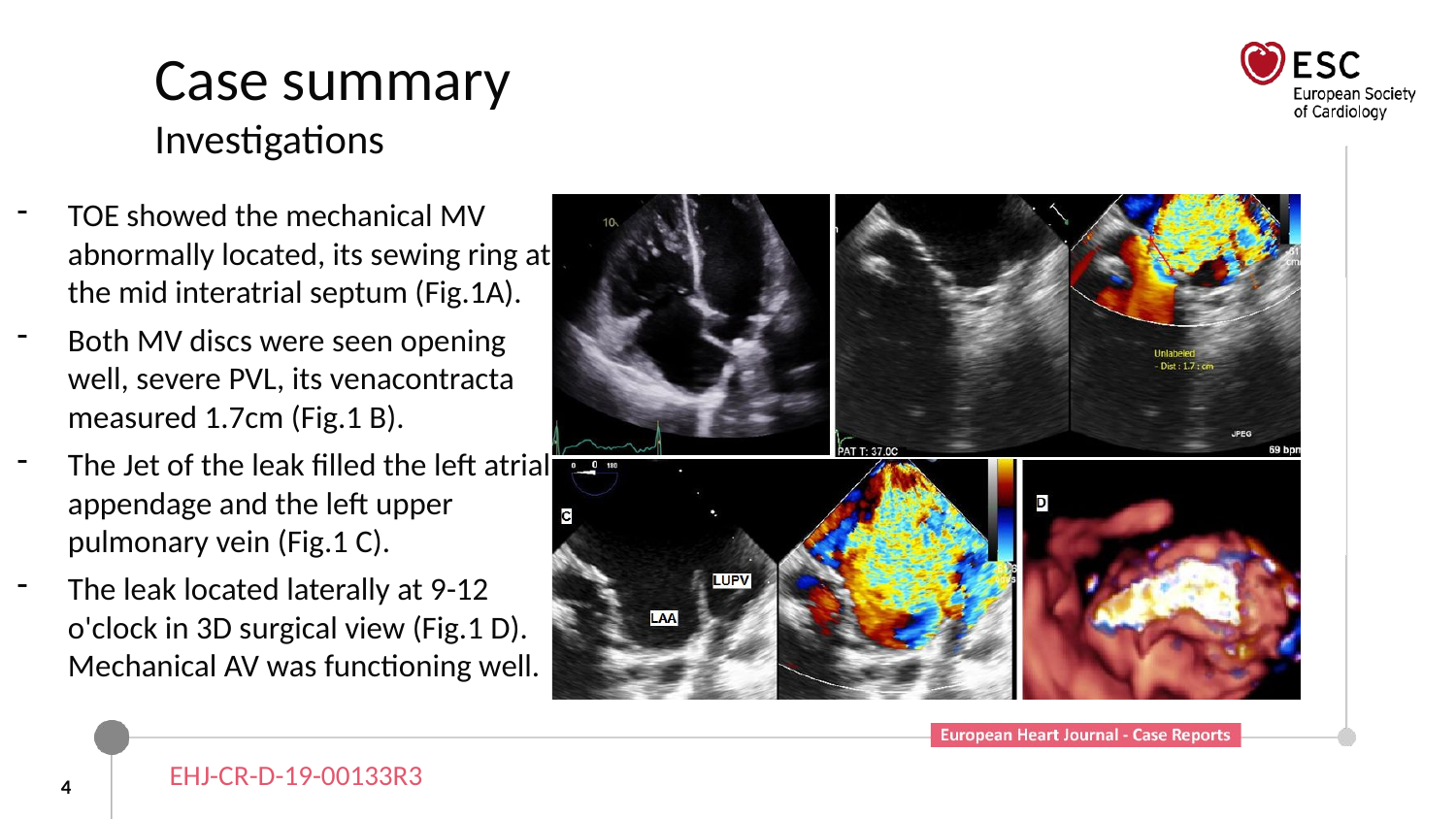

# Case summary Investigations
TOE showed the mechanical MV abnormally located, its sewing ring at the mid interatrial septum (Fig.1A).
Both MV discs were seen opening well, severe PVL, its venacontracta measured 1.7cm (Fig.1 B).
The Jet of the leak filled the left atrial appendage and the left upper pulmonary vein (Fig.1 C).
The leak located laterally at 9-12 o'clock in 3D surgical view (Fig.1 D). Mechanical AV was functioning well.
4
EHJ-CR-D-19-00133R3

## Slide 5
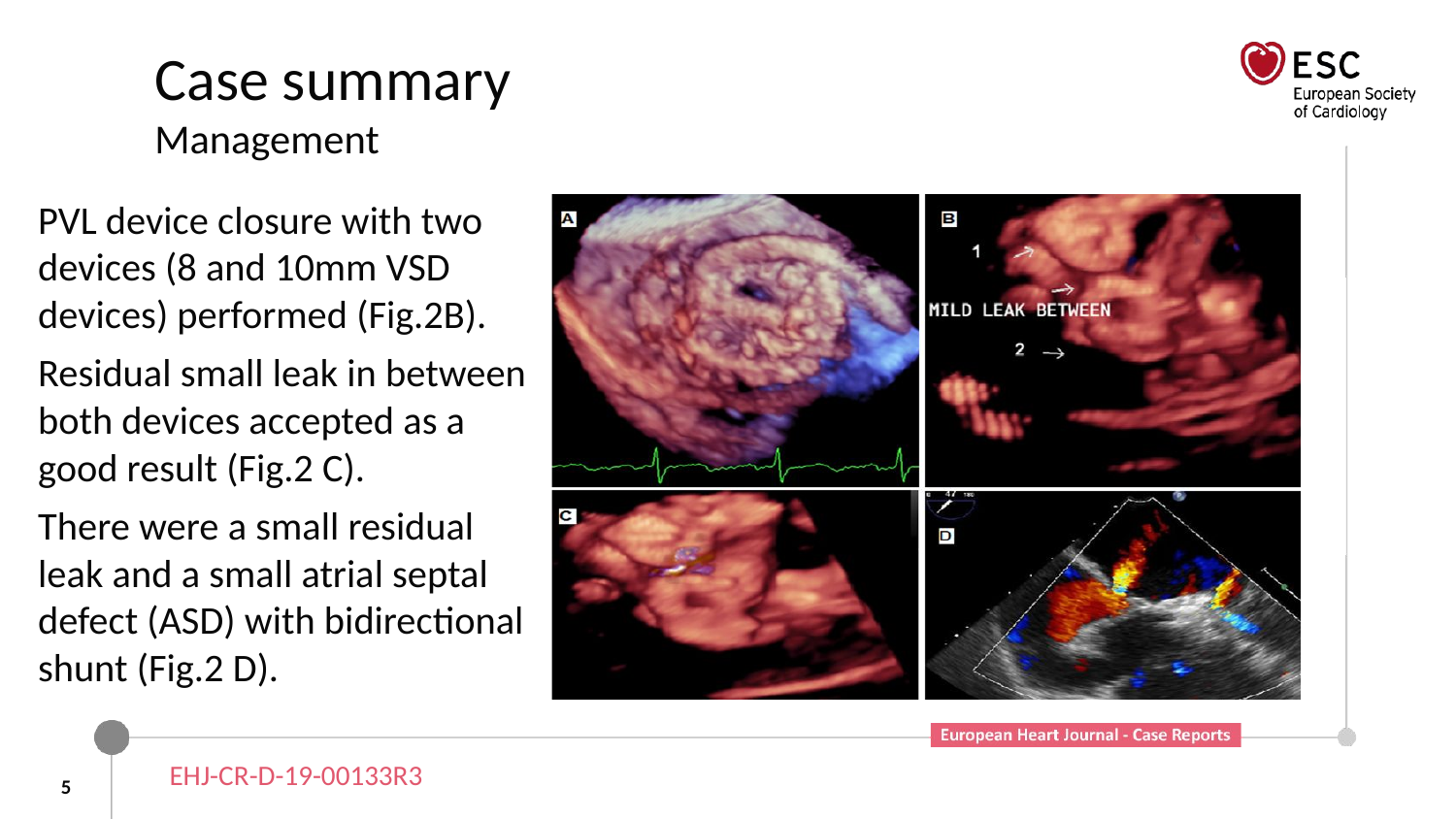

# Case summary Management
PVL device closure with two devices (8 and 10mm VSD devices) performed (Fig.2B).
Residual small leak in between both devices accepted as a good result (Fig.2 C).
There were a small residual leak and a small atrial septal defect (ASD) with bidirectional shunt (Fig.2 D).
5
EHJ-CR-D-19-00133R3

## Slide 6
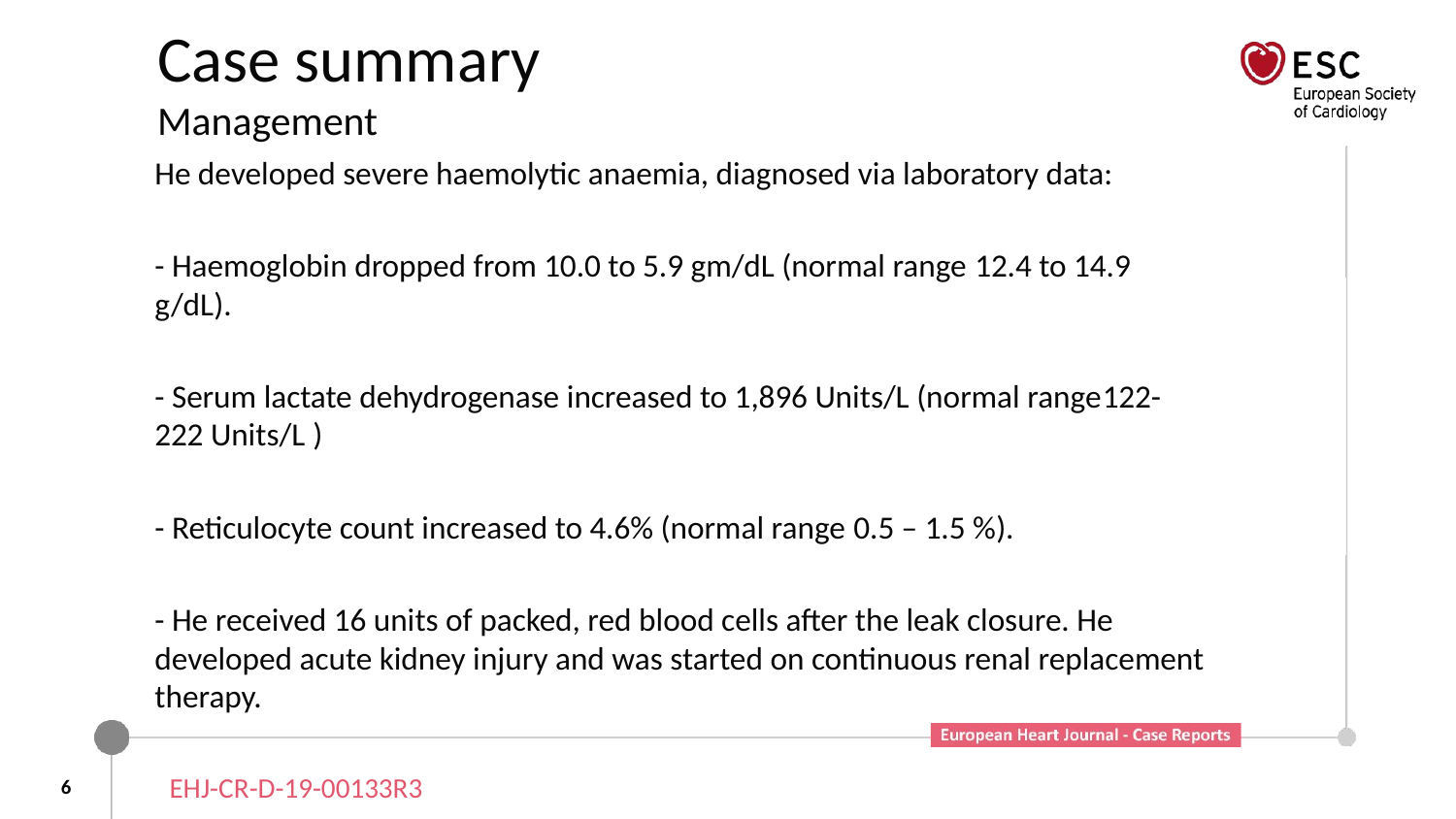

Case summary
Management
#
He developed severe haemolytic anaemia, diagnosed via laboratory data:
- Haemoglobin dropped from 10.0 to 5.9 gm/dL (normal range 12.4 to 14.9 g/dL).
- Serum lactate dehydrogenase increased to 1,896 Units/L (normal range122-222 Units/L )
- Reticulocyte count increased to 4.6% (normal range 0.5 – 1.5 %).
- He received 16 units of packed, red blood cells after the leak closure. He developed acute kidney injury and was started on continuous renal replacement therapy.
6
EHJ-CR-D-19-00133R3

## Slide 7
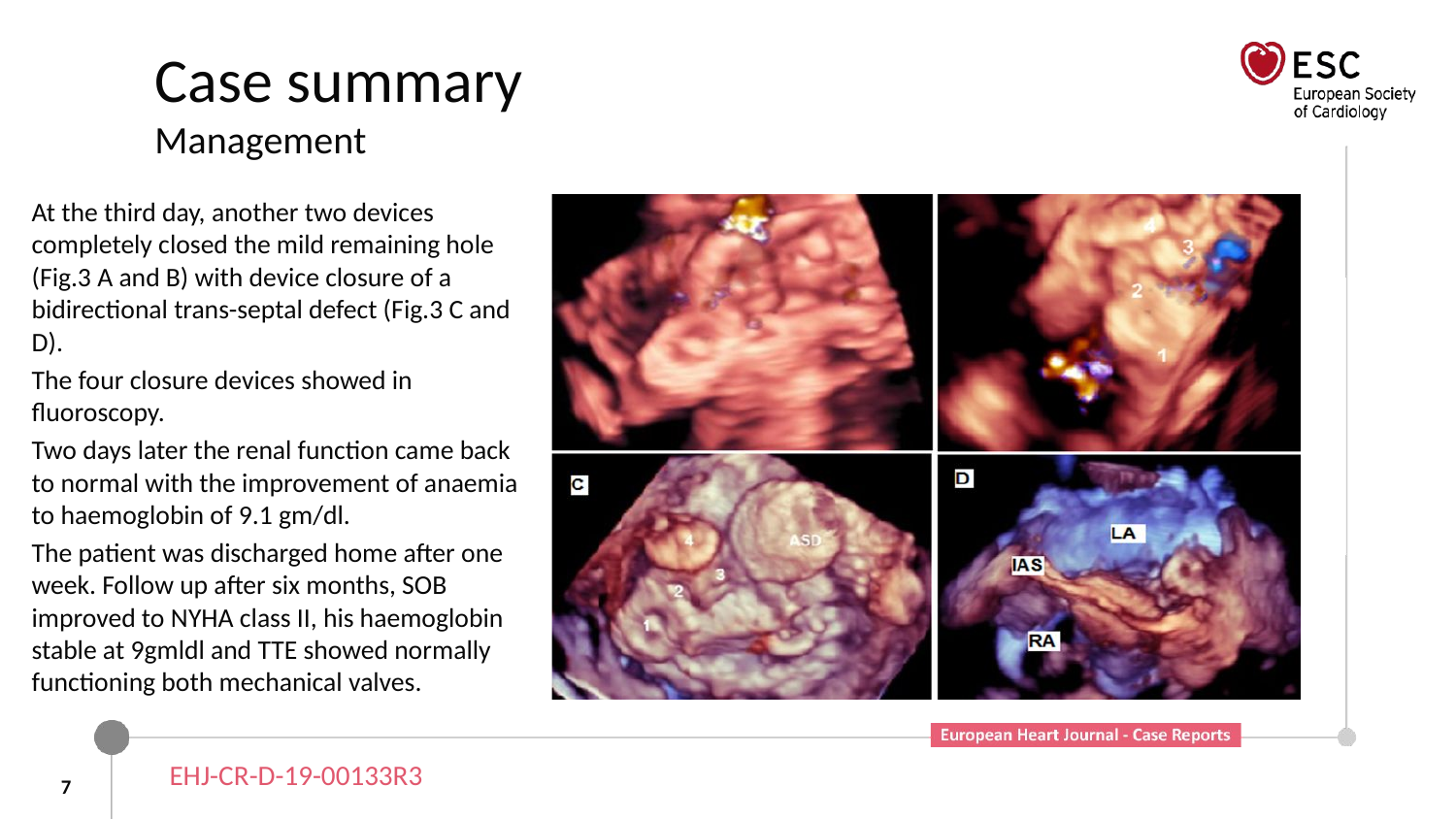

# Case summary Management
At the third day, another two devices completely closed the mild remaining hole (Fig.3 A and B) with device closure of a bidirectional trans-septal defect (Fig.3 C and D).
The four closure devices showed in fluoroscopy.
Two days later the renal function came back to normal with the improvement of anaemia to haemoglobin of 9.1 gm/dl.
The patient was discharged home after one week. Follow up after six months, SOB improved to NYHA class II, his haemoglobin stable at 9gmldl and TTE showed normally functioning both mechanical valves.
7
EHJ-CR-D-19-00133R3

## Slide 8
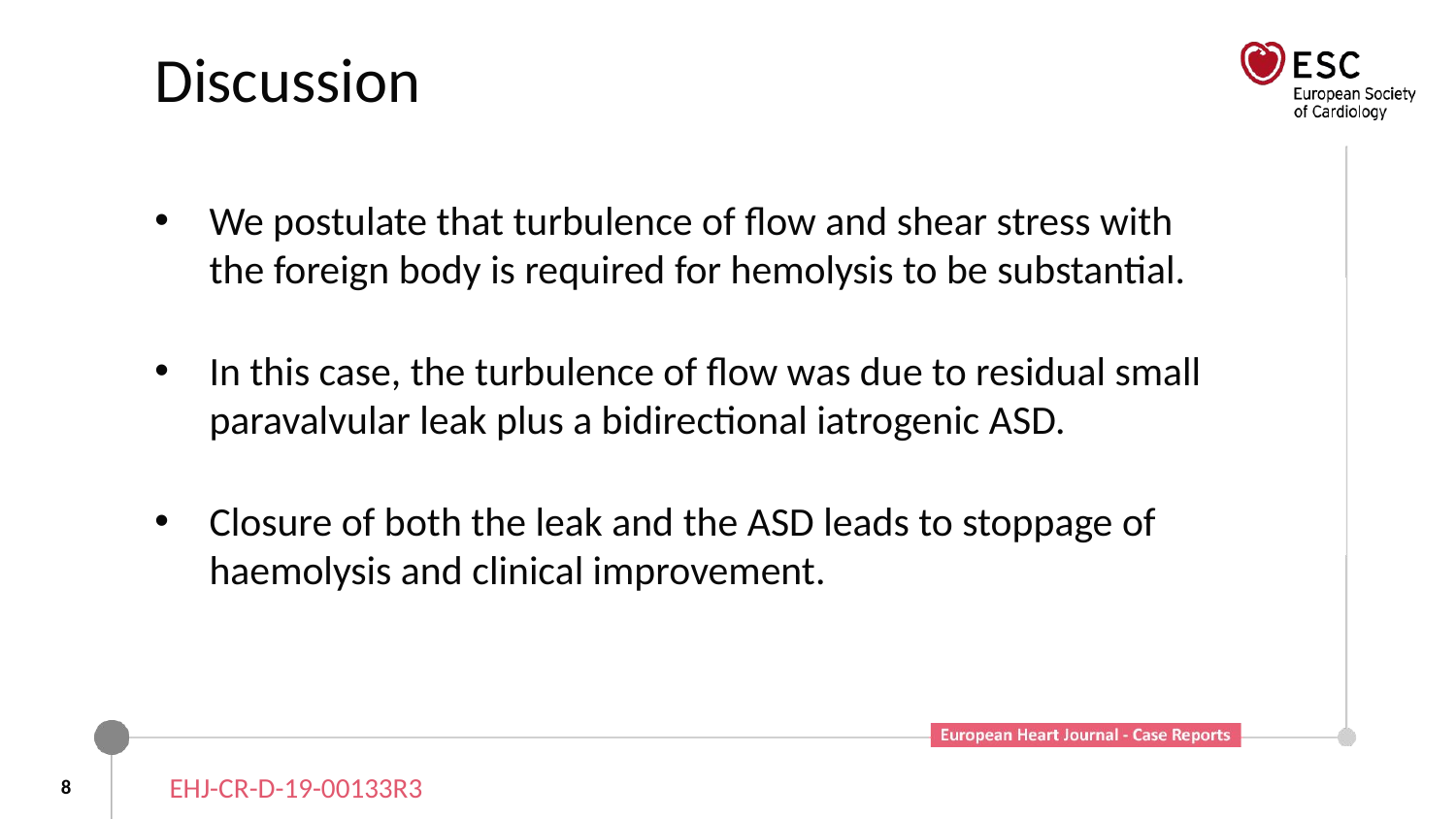

# Discussion
We postulate that turbulence of flow and shear stress with the foreign body is required for hemolysis to be substantial.
In this case, the turbulence of flow was due to residual small paravalvular leak plus a bidirectional iatrogenic ASD.
Closure of both the leak and the ASD leads to stoppage of haemolysis and clinical improvement.
8
EHJ-CR-D-19-00133R3

## Slide 9
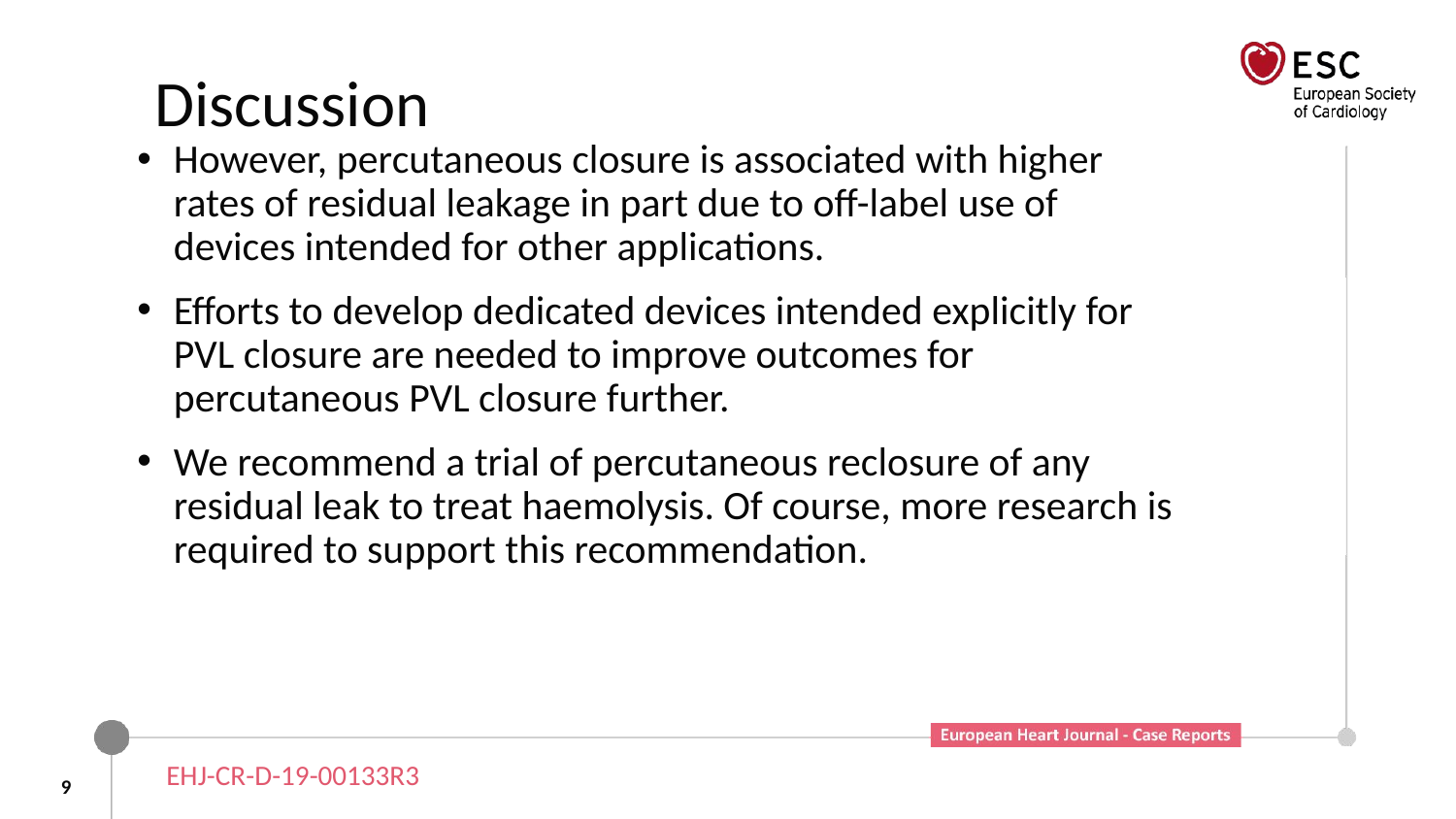

# Discussion
However, percutaneous closure is associated with higher rates of residual leakage in part due to off-label use of devices intended for other applications.
Efforts to develop dedicated devices intended explicitly for PVL closure are needed to improve outcomes for percutaneous PVL closure further.
We recommend a trial of percutaneous reclosure of any residual leak to treat haemolysis. Of course, more research is required to support this recommendation.
EHJ-CR-D-19-00133R3
9

## Slide 10
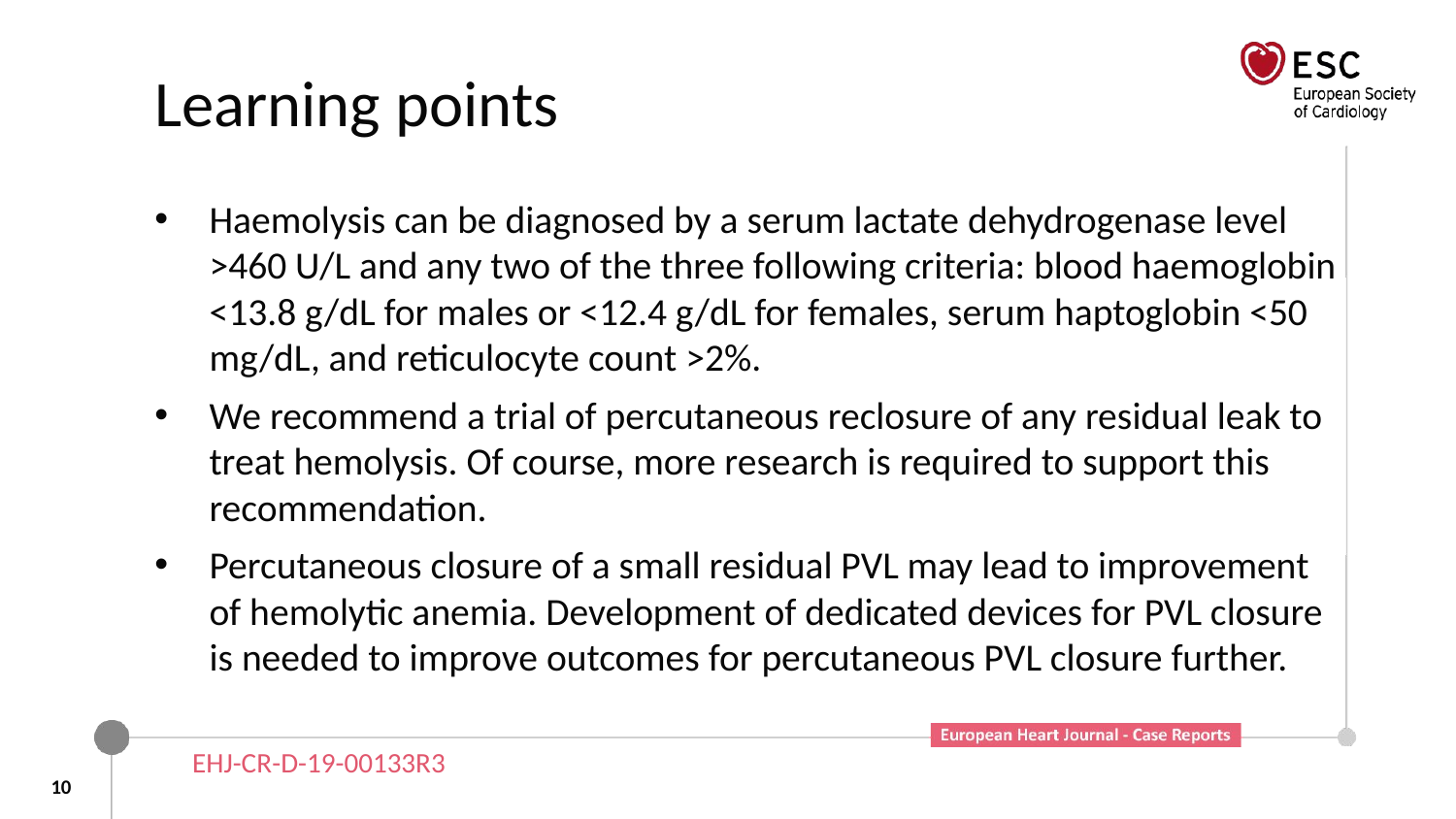

# Learning points
Haemolysis can be diagnosed by a serum lactate dehydrogenase level >460 U/L and any two of the three following criteria: blood haemoglobin <13.8 g/dL for males or <12.4 g/dL for females, serum haptoglobin <50 mg/dL, and reticulocyte count >2%.
We recommend a trial of percutaneous reclosure of any residual leak to treat hemolysis. Of course, more research is required to support this recommendation.
Percutaneous closure of a small residual PVL may lead to improvement of hemolytic anemia. Development of dedicated devices for PVL closure is needed to improve outcomes for percutaneous PVL closure further.
EHJ-CR-D-19-00133R3
10

## Slide 11
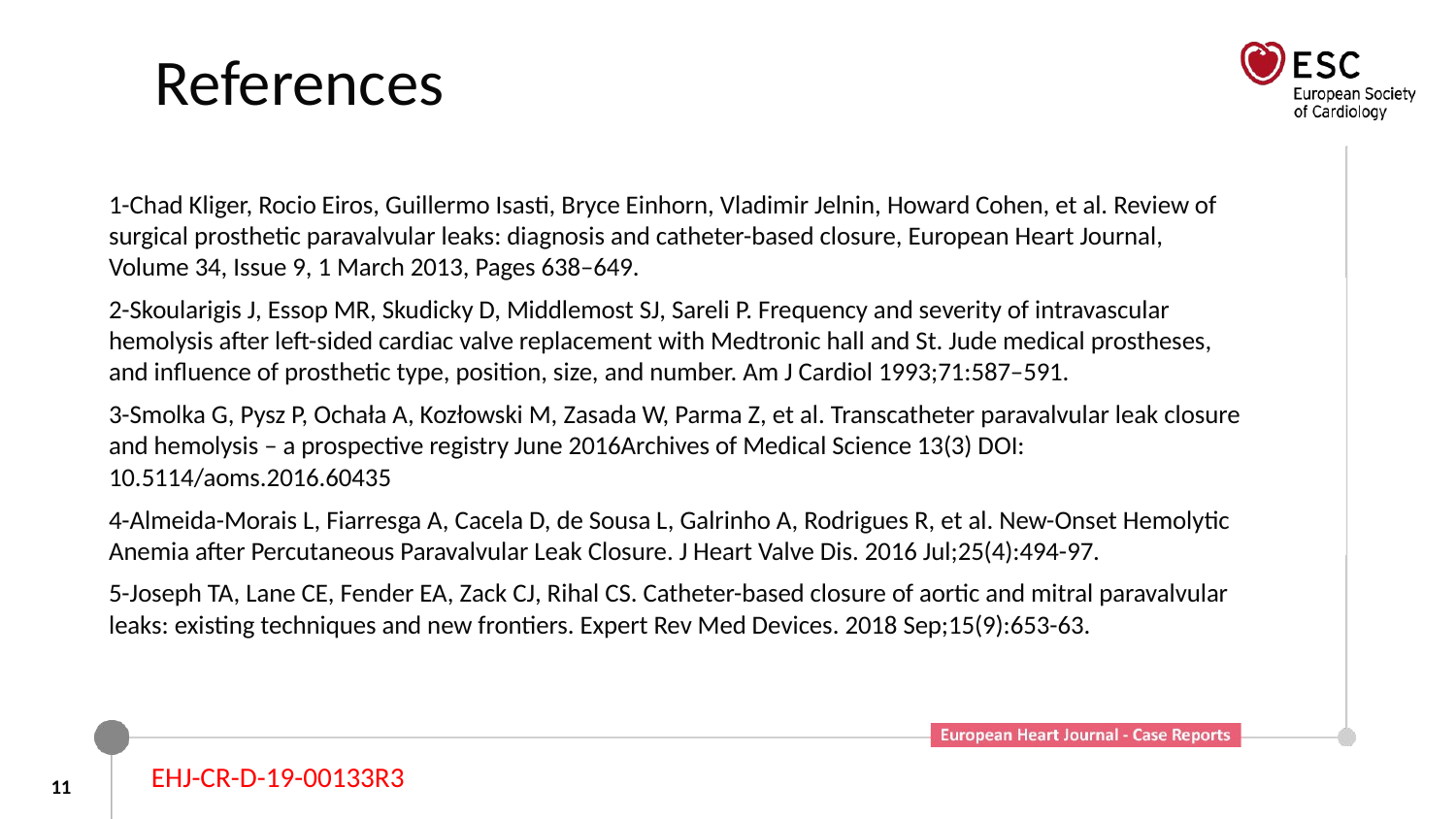

# References
1-Chad Kliger, Rocio Eiros, Guillermo Isasti, Bryce Einhorn, Vladimir Jelnin, Howard Cohen, et al. Review of surgical prosthetic paravalvular leaks: diagnosis and catheter-based closure, European Heart Journal, Volume 34, Issue 9, 1 March 2013, Pages 638–649.
2-Skoularigis J, Essop MR, Skudicky D, Middlemost SJ, Sareli P. Frequency and severity of intravascular hemolysis after left-sided cardiac valve replacement with Medtronic hall and St. Jude medical prostheses, and influence of prosthetic type, position, size, and number. Am J Cardiol 1993;71:587–591.
3-Smolka G, Pysz P, Ochała A, Kozłowski M, Zasada W, Parma Z, et al. Transcatheter paravalvular leak closure and hemolysis – a prospective registry June 2016Archives of Medical Science 13(3) DOI: 10.5114/aoms.2016.60435
4-Almeida-Morais L, Fiarresga A, Cacela D, de Sousa L, Galrinho A, Rodrigues R, et al. New-Onset Hemolytic Anemia after Percutaneous Paravalvular Leak Closure. J Heart Valve Dis. 2016 Jul;25(4):494-97.
5-Joseph TA, Lane CE, Fender EA, Zack CJ, Rihal CS. Catheter-based closure of aortic and mitral paravalvular leaks: existing techniques and new frontiers. Expert Rev Med Devices. 2018 Sep;15(9):653-63.
EHJ-CR-D-19-00133R3
11
